# Clinical characteristics and treatment outcomes of adult acute promyelocytic leukemia in the West Bank of Palestine: a single center retrospective study

**DOI:** 10.1186/s12885-026-16031-0

**Published:** 2026-04-20

**Authors:** Raghad Tanbour, Manal Ishtayeh, Mohammad Khaled Alhindi, Riad Amer

**Affiliations:** 1 Department of Internal Medicine, Arab American University, Jenin, Palestinian Territory; 2 Department of Internal Medicine, An-Najah National university Hospital, Nablus, Palestinian Territory; 3 Department of Hemato-oncology, An-Najah National University Hospital, Nablus, Palestinian Territory

**Keywords:** Acute promyelocytic leukemia, Induction therapy, Eearly mortality, Complete remission, Arsenic trioxide, Palestine

## Abstract

**Background:**

Acute promyelocytic leukemia (APL), a distinct subtype of acute myeloid leukemia (AML), accounts for 5–20% of AML and carries a high risk of early, often hemorrhagic, mortality related to disseminated intravascular coagulation (DIC). With all-trans retinoic acid (ATRA) and arsenic trioxide (ATO), APL has become highly curable, with survival approaching 90%. Data from Palestine are limited. This study addresses an important regional data gap by describing the presentation, complications, and outcomes of adults with APL treated at a tertiary Palestinian cancer center and by comparing patients with and without early death.

**Methods:**

We retrospectively reviewed 30 adults (≥18 years) diagnosed with APL at An-Najah National University Hospital (NNUH), West Bank, Palestine, between January 2016 and June 2024. Patients were classified as standard-risk (WBC ≤10×10⁹/L) or high-risk (WBC >10×10⁹/L). Standard-risk patients received ATRA+ATO induction, whereas high-risk patients received ATRA plus anthracycline-based induction. Consolidation with ATRA+ATO was given to patients who proceeded to consolidation in both groups. Early death was defined as death within 30 days of diagnosis.

**Results:**

The cohort included 15 males and 15 females, with a median age of 37 years (range, 18–79 years); 22 (73.3%) were standard-risk and 8 (26.7%) were high-risk. Median baseline values were hemoglobin 9.0 g/dL, WBC 4.8×10⁹/L, platelets 18.1×10⁹/L, ANC 0.95×10⁹/L, PT 17.2 s, aPTT 27.45 s, fibrinogen 142.5 mg/dL, and D-dimer 30.9 mg/L FEU. Early death occurred in 13.3% (4/30) and was hemorrhage-related in the setting of coagulopathy/DIC. All patients who survived beyond 30 days achieved complete hematologic remission. Kaplan–Meier overall survival was 96.4% at 1 year and 87.7% at 2 years; median overall survival was 56.2 months (95% CI, 33.6–78.9). Compared with patients without early death, those with early death had higher admission WBC counts, longer aPTT, and more frequent ICU admission.

**Conclusions:**

In this single-center Palestinian cohort, ATRA-based treatment yielded high remission rates and no observed relapse during follow-up. Early mortality remained the main barrier to cure and occurred in patients presenting with a higher leukocyte burden and evidence of more severe coagulopathy. Prompt diagnosis, immediate ATRA initiation, and intensive supportive care remain essential in resource-limited settings.

## Background

APL is a hematologic emergency because of severe coagulopathy and the risk of early fatal hemorrhage [1]. Immediate ATRA, risk-adapted therapy (often including ATO), and intensive transfusion support have markedly improved outcomes, but early deaths still occur, especially in resource-limited settings [2]. In the West Bank, cancer is the second leading cause of death, and hematologic malignancies such as APL face unique challenges related to fragmented cancer care, delays in diagnosis and referral, restricted access to intensive supportive care (including blood products and intensive care beds), and occasional constraints in the availability of ATRA and ATO [3]. APL care in the West Bank is concentrated in two primary facilities. An-Najah National University Hospital (NNUH) serves as a key referral center for hematologic malignancies, managing cases from both the West Bank and the Gaza Strip. The second facility providing specialized care for these patients is Augusta Victoria Hospital.

Despite notable advances in APL treatment worldwide, data describing the clinical profile, treatment patterns, and outcomes of Palestinian patients remain limited. This study sought to characterize the clinical features and treatment outcomes of adults with APL treated at NNUH and to describe differences between patients with and without early death.

## Methods

### Study design and setting

This was a retrospective cohort analysis conducted at An-Najah National University Hospital (NNUH), a tertiary care cancer center in Nablus, West Bank, Palestine. We examined the electronic medical records of adult patients (aged ≥ 18 years) diagnosed with APL between January 2016 and June 2024 and treated with curative intent. Data were collected on demographics, clinical and laboratory characteristics at diagnosis, treatment regimens, treatment-related complications, remission status, relapse, and survival.

### Study population and sample size

All consecutive adults with a confirmed diagnosis of APL who were treated at NNUH during the study period were eligible. During the study period, 187 adults were evaluated for acute myeloid leukemia at NNUH, and 30 were diagnosed with APL (16.0% of AML). All 30 patients met the eligibility criteria and were included; no patients were excluded because of missing data. A complete denominator of all AML cases managed during the study period was not available from a single registry because some suspected AML cases were treated elsewhere or died before referral; therefore, we did not estimate the proportion of APL among all AML diagnoses.

### Inclusion and exclusion criteria

Inclusion criteria were: (1) age ≥ 18 years; (2) diagnosis of APL confirmed by bone marrow morphology, immunophenotyping, and cytogenetic and/or molecular studies on bone marrow samples, including conventional karyotyping and/or fluorescence in situ hybridization (FISH) for PML–RARA; and (3) initiation of APL-directed therapy at NNUH. Patients referred from other centers were included if confirmatory testing and clinical data were available or were repeated at NNUH.

Diagnosis of APL was based on the World Health Organization (WHO) classification [4]. DIC was defined according to the International Society on Thrombosis and Haemostasis (ISTH) scoring system, with a score ≥ 5 indicating overt DIC [5]. Coagulopathy was defined as prolonged PT and/or aPTT and/or decreased fibrinogen levels. Because documentation of all ISTH criteria was incomplete, overt DIC could not be verified consistently in this retrospective review; therefore, patients documented as having “DIC” or “coagulopathy” were classified as having coagulopathy in the analysis.

## Treatment protocols

All patients received risk-adapted induction therapy aligned with contemporary international recommendations [6]. Per the updated Sanz score, patients with low- or intermediate-risk disease were grouped as standard-risk (WBC ≤ 10 × 10^9/L) and those with high-risk disease as high-risk (WBC > 10 × 10^9/L) [7]. Standard-risk patients received ATRA 45 mg/m^2/day plus ATO 0.15 mg/kg/day. High-risk patients received ATRA in combination with anthracycline-based chemotherapy (daunorubicin or idarubicin) [6]; in one patient, cytarabine plus mitoxantrone was used during induction only based on treating physician choice and local drug availability. ATRA was initiated immediately when APL was clinically suspected, without waiting for confirmatory cytogenetic or molecular testing.

Supportive care included platelet transfusions (target > 30–50 × 10⁹/L), cryoprecipitate to maintain fibrinogen ≥ 100 mg/dL, and fresh frozen plasma for prolonged PT/INR (target INR ≤ 1.5, per institutional practice). ATRA and ATO were temporarily suspended in cases of severe complications and reintroduced once clinical improvement was observed.

All patients who proceeded to consolidation were planned to receive four cycles of ATRA plus ATO. Maintenance therapy (intermittent ATRA with 6-mercaptopurine and methotrexate for up to 2 years) was used selectively, mainly with chemotherapy-based approaches, and was not routinely given after ATRA + ATO-based programs. After consolidation, molecular assessment for PML–RARA was performed using FISH. Because of resource constraints, standardized minimal residual disease monitoring by RT-PCR was not consistently performed; access was limited and documentation was incomplete.

### Data collection

Data were retrieved from electronic medical records using a standardized data collection form, including demographic, clinical, laboratory, treatment, and outcome variables. D-dimer was reported as mg/L FEU. Early death was defined as death from any cause within 30 days of APL diagnosis.

Hematologic complete remission (CR) was defined as ANC ≥ 1.5 × 10⁹/L, platelet count > 100 × 10⁹/L, normocellular bone marrow with < 5% blasts plus promyelocytes, and absence of clinical evidence of APL.

Patients were followed from diagnosis until death, last documented contact, or 30 June 2024, whichever occurred first. Analyses were carried out on a variable-by-variable basis when data were missing.

### Ethics

This study received ethical approval from the Ethics Committee of An-Najah National University Hospital, Nablus, Palestine, in 2023, in line with national regulations and the principles of the Declaration of Helsinki (1964) and its subsequent amendments [8]. Given the retrospective design and use of anonymized data, the requirement for informed consent was waived.

### Statistical analysis

Given the small sample size and limited number of early-death events, multivariable modeling was not performed. Statistical analyses were conducted using SPSS version 25. Continuous variables were evaluated for normality and are summarized as medians with interquartile ranges (IQRs) because most variables were not normally distributed, whereas categorical variables are summarized as frequencies and percentages. For descriptive comparisons between patients with early death (≤ 30 days from diagnosis) and those without early death, continuous variables were compared using the Mann–Whitney U test and categorical variables using the chi-square test or Fisher’s exact test, as appropriate. Because only four early-death events were observed, multivariable regression analyses were not performed, and terms implying independent association or prediction were avoided. Two-sided p-values < 0.05 were considered statistically significant. Overall survival was estimated using the Kaplan–Meier method and compared between groups using the log-rank test; survival times are presented as medians.

## Results

### Clinical presentation and baseline characteristics

Thirty adults with newly diagnosed APL were included. The cohort comprised 15 males (50.0%) and 15 females (50.0%), with a median age at diagnosis of 37 years (range, 18–79 years). Approximately one-third of patients (33.3%) were residents of the Gaza Strip, and the remainder were mainly from the northern West Bank.

Most patients had good performance status: 20 (66.7%) had ECOG 0, 8 (26.7%) had ECOG 1, and 2 (6.7%) had ECOG 2. Using the WBC-based risk grouping commonly applied in current treatment algorithms, 22 patients (73.3%) were standard-risk (WBC ≤ 10 × 10⁹/L) and 8 (26.7%) were high-risk (WBC > 10 × 10⁹/L).

Baseline laboratory values showed pronounced cytopenias and coagulopathy. Median baseline values at diagnosis were hemoglobin 9.0 g/dL, WBC 4.8 × 10⁹/L, platelets 18.1 × 10⁹/L, ANC 0.95 × 10⁹/L, PT 17.2 s, aPTT 27.45 s, fibrinogen 142.5 mg/dL, and D-dimer 30.9 mg/L FEU. Bleeding was the most common presenting symptom (30.0%), followed by fever (10.0%) and fatigue (7.0%).

### Induction therapy, complications, and early mortality

ATRA 45 mg/m²/day was initiated promptly when APL was clinically suspected. Risk-adapted induction therapy included ATRA + ATO for standard-risk patients and ATRA plus anthracycline-based chemotherapy for high-risk patients.

One patient (3.3%) died of fulminant hemorrhage before induction therapy could be started. Among the remaining 29 patients who initiated induction, 16 (55.2%) received hydroxyurea cytoreduction before or during induction, and one high-risk patient received cytarabine cytoreduction before induction because hydroxyurea was unavailable. Induction therapy was ATO-based (ATRA + ATO) in 21/29 (72.4%) and ATRA plus chemotherapy without ATO in 8/29 (27.6%). The median time to hematologic remission was 35 days (IQR, 30–42 days). No patient required a second induction course; in several cases, the time to remission reflected prolonged cytopenia and delayed count recovery, consistent with differentiation-based regimens and intensive supportive care.

Patients who received hydroxyurea cytoreduction had significantly higher baseline WBC counts compared with those who did not (*p* = 0.002), and cytoreduction was used more frequently among high-risk patients (*p* = 0.039). Bleeding complications during induction were numerically more frequent in the cytoreduction group (9/16) compared with patients not receiving cytoreduction (4/14), although this difference was not statistically significant (*p* = 0.159). Early death (≤ 30 days) was observed in 2/16 patients in the cytoreduction group versus 2/14 in the no-cytoreduction group (*p* = 1.000).

Complications during induction were observed in 13 patients (43.3%). Sepsis and DIC with bleeding were the most frequent complications.

Differentiation syndrome (ATRA syndrome) was documented in 7/29 patients (24.1%). All cases were managed with dexamethasone; ATRA was temporarily interrupted and then resumed when clinically appropriate. When stratified by induction regimen, 4/21 (19.0%) patients treated with ATRA + ATO-based regimens developed differentiation syndrome versus 3/8 (37.5%) of those treated with ATRA+chemotherapy-based regimens. The difference in incidence between the two groups was not statistically significant (*p* = 0.64). The severity of differentiation syndrome could not be graded because detailed documentation was not consistently available in the medical records.

Intensive care unit (ICU) admission was observed in 7 patients (23.3%). Of these, 5 died: 1 before induction, 3 during induction, and 1 during consolidation.

Overall, four patients (13.3%) experienced early death (≤ 30 days from diagnosis): one before starting induction and three during induction. All early deaths were attributable to hemorrhagic complications occurring in the context of coagulopathy/DIC and were often accompanied by profound thrombocytopenia. Descriptive comparisons between patients with early death (≤ 30 days; *n* = 4) and those without early death (*n* = 26) are summarized in Table 1. Patients with early death had a higher median admission WBC count (28.41 vs. 3.72 × 10⁹/L; *p* = 0.026) and longer median aPTT (30.25 vs. 26.80 s; *p* = 0.026). ICU admission was also more frequent among patients with early death (4/4 [100.0%] vs. 3/25 [12.0%]; *p* = 0.001). No statistically significant differences were observed for age, PT, fibrinogen, sex, cytoreduction before induction, bleeding at presentation, or ATRA use. Because of the small number of early-death events, multivariable modeling was not performed.

**Table 1:** Descriptive comparison between patients with and without early death (≤ 30 days). Continuous variables are presented as median (IQR); categorical variables are presented as n (%)

Variable	No early death (*n* = 26)	Early death (*n* = 4)	*p*-value
Age, years, median (IQR)	36.5 (25.0)	37.5 (14.0)	0.976
WBC on admission, x10^9/L, median (IQR)	3.715 (13.63)	28.410 (51.00)	0.026
PT, sec, median (IQR)	16.3 (3.68)	21.5 (7.88)	0.576
aPTT, sec, median (IQR)	26.8 (3.15)	30.25 (2.90)	0.026
Fibrinogen, mg/dL, median (IQR)	150.5 (155.25)	98.0 (77.25)	0.123
Female sex, n (%)	12/26 (46.2%)	3/4 (75.0%)	0.598
Cytoreduction before induction, n (%)	14/26 (53.8%)	2/4 (50.0%)	1.000
Bleeding at presentation, n (%)	10/26 (38.5%)	3/4 (75.0%)	0.290
ICU admission, n (%)	3/25 (12.0%)	4/4 (100.0%)	0.001
ATRA use, n (%)	7/26 (26.9%)	0/4 (0.0%)	0.548

Of the 30 patients, 26 (86.7%) survived induction and proceeded to consolidation therapy. Consolidation was predominantly ATRA plus ATO and was completed at our center by most standard-risk patients (18/20, 90.0%); a minority (*n* = 2) continued treatment at another center. One patient died during consolidation.

At last follow-up, 22/30 patients (73.3%) were alive; follow-up was administratively censored on 30 June 2024 (median follow-up by reverse Kaplan-Meier, 38.0 months).

After consolidation, remission assessment by bone marrow PML-RARA FISH was available for 17 patients and was negative in all cases. Because access to PML-RARA RT-PCR was limited and documentation was incomplete, the exact number of patients who underwent PCR-based assessment could not be reliably determined retrospectively; however, among those with recorded RT-PCR results, all were negative. Maintenance therapy (ATRA + 6-MP + MTX) was initiated in 13 patients (9 standard-risk and 4 high-risk), with 8 completing the planned 2-year course through 30/6/2024; maintenance was used mainly in patients treated with chemotherapy-based regimens and selectively based on clinician preference for standard-risk patients. Three additional patients died after completing maintenance therapy from a non-APL-related cause. No hematologic or molecular relapses were detected during follow-up, and none of the patients underwent hematopoietic stem cell transplantation.

**Fig. 1:**
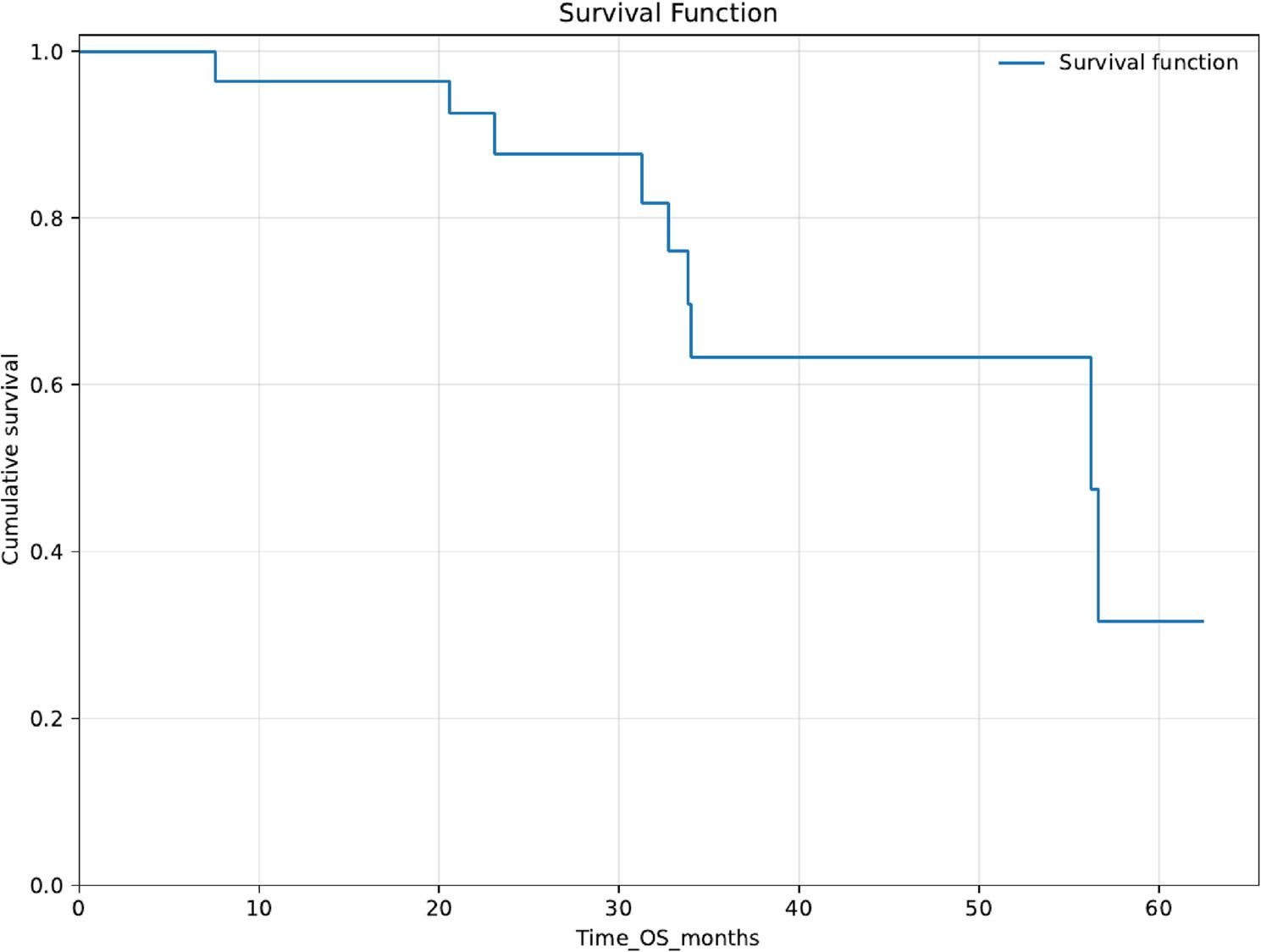
Kaplan–Meier overall survival (OS). Kaplan–Meier estimate of OS for the entire cohort. Death was the event; patients were censored at the administrative end of follow-up (30 June 2024)

Overall survival (OS) was estimated using Kaplan–Meier methods with death as the event and administrative censoring at 30 June 2024. In the full cohort (*n* = 30), median OS was 56.209 months (95% CI, 33.561–78.857). Kaplan–Meier estimates indicated high early survival, with OS of 96.4% at 1 year and 87.7% at 2 years. The Kaplan–Meier overall survival curve is shown in Fig. 1.

**Fig. 2:**
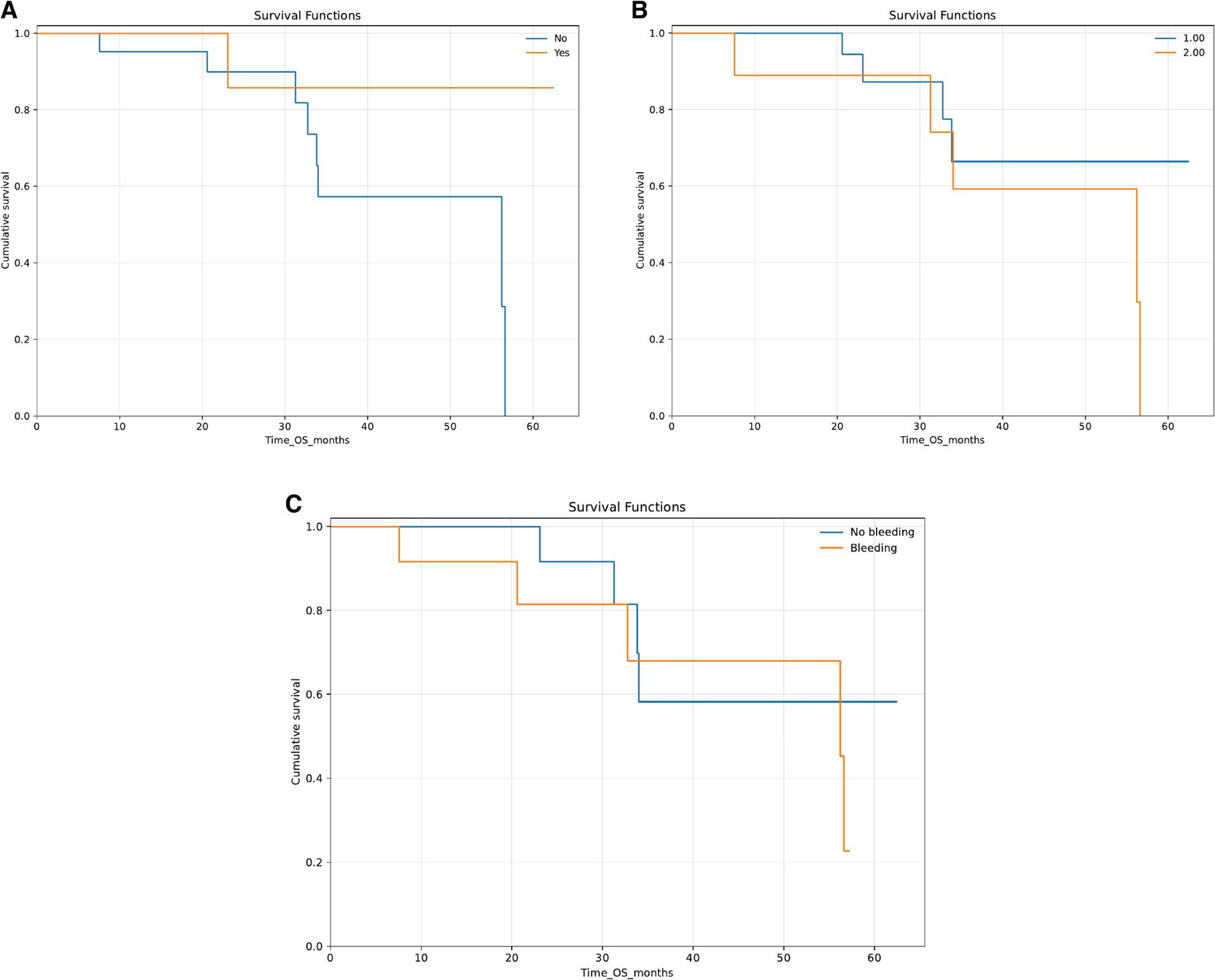
Kaplan-Meier overall survival by selected clinical variables. **A**. Pre-induction cytoreduction (hydroxyurea). **B**. Induction regimen (ATRA+ATO vs ATRA+chemotherapy). **C**. Bleeding at presentation. **A**: OS stratified by pre-induction cytoreduction (hydroxyurea). **B**: OS stratified by induction regimen (ATRA+ATO vs ATRA+chemotherapy). **C**: OS stratified by bleeding at presentation; in panel 2C, the blue line represents patients without bleeding at presentation and the orange line represents patients with bleeding at presentation

When stratified by pre-induction cytoreduction, OS did not differ significantly between patients who received hydroxyurea and those who did not (log-rank *p* = 0.254). Survival by pre-induction cytoreduction is shown in Fig. 2A.

When stratified by induction regimen, deaths were observed in 4/21 patients treated with an ATRA + ATO backbone compared with 5/9 treated with ATRA+chemotherapy. Overall survival appeared higher in patients treated with ATRA + ATO than in those treated with ATRA+chemotherapy, but the difference was not statistically significant (log-rank *p* = 0.260) and should be interpreted cautiously given the limited sample size and event count. Survival by induction backbone is shown in Fig. 2B.

In this 8-year retrospective cohort of adults with APL treated at a single tertiary center in Palestine, risk-adapted ATRA-based therapy was associated with high rates of hematologic remission and no documented relapse during follow-up. Although the study does not offer therapeutic novelty, it provides rare real-world adult Palestinian data on APL presentation, supportive care, and outcomes.

Our findings support the feasibility of implementing contemporary APL protocols in a resource-constrained environment [9, 10]. Although late relapse cannot be excluded given the limited sample size and heterogeneous follow-up, the absence of observed relapse and the high remission rate are consistent with international data demonstrating the curability of APL with differentiation therapy, particularly ATRA combined with ATO, compared with classical chemotherapy-based regimens [11–13].

The median age of 37 years and balanced sex distribution in our cohort are consistent with reports from several other regions but younger than in some series, including a Mexican multicenter study and a population-based study from Hong Kong [14, 15]. Most of our patients presented with good performance status (ECOG 0–1), in line with previous studies indicating that most patients with APL fall between ECOG 0 and 2 at diagnosis [16, 17].

Bleeding at presentation and coagulopathy/DIC in about one-quarter of patients underscore the well-recognized coagulopathy associated with APL, which remains a major driver of early mortality [18]. The hemorrhagic diathesis in APL is multifactorial, involving DIC, hyperfibrinolysis, and severe thrombocytopenia [19]. In our cohort, all early deaths were hemorrhagic events in the setting of coagulopathy/DIC. The descriptive between-group differences observed for higher WBC and longer aPTT at diagnosis are biologically plausible and likely reflect the close relationship between disease burden and the severity of coagulopathy; given the small number of events, these findings should be interpreted cautiously.

Overall survival in this cohort was encouraging, with a median OS of 56.2 months and Kaplan–Meier estimates of 96.4% and 87.7% at 1 and 2 years, respectively, supporting the high curability of APL when contemporary ATRA-based therapy and supportive care are delivered. Internationally, outcomes in modern ATRA + ATO clinical trials are typically even higher (for example, approximately 97% 2-year event-free survival in ATRA + ATO arms), whereas population-based real-world series report lower survival largely because early death remains substantial [11, 20–22]. In developing-setting networks such as IC-APL, 2-year OS of around 80% has been reported, placing our 2-year OS (87.7%) within the range observed outside randomized trials [9]. A Mexican single-center experience using a modified IC-APL protocol also emphasized that protocol adherence, supportive care, and organized care pathways can reproduce favorable outcomes in resource-constrained settings [23]. Although we explored OS differences by bleeding at presentation, pre-induction cytoreduction, and induction backbone, none of these comparisons reached statistical significance, which is likely related to limited power given the small sample size and low number of events. Larger multicenter cohorts with standardized follow-up and time-to-event documentation are needed to determine whether regimen choice or early clinical features independently influence survival in similar resource-constrained settings.

Differentiation syndrome was observed in 24.1% of patients and occurred with both ATO-based and chemotherapy-based induction. All documented cases responded to dexamethasone, and ATRA was temporarily interrupted when clinically indicated; however, severity could not be graded because documentation was incomplete. Cytoreductive strategies were used when leukocytosis was present at baseline or increased during induction. Hydroxyurea was preferentially given to patients with higher baseline leukocytosis and higher-risk disease, and its use was not associated with increased early death, although bleeding complications were numerically more frequent among patients receiving cytoreduction, likely reflecting confounding by baseline disease burden.

High-risk disease accounted for 26.7% of the cohort. Referral barriers may have influenced the observed risk distribution. Patients with hyperleukocytosis and severe coagulopathy are more likely to deteriorate rapidly, and those facing delays in transfer—particularly from Gaza—may not have survived long enough to be captured in our dataset. Therefore, the proportion of high-risk cases in the overall Palestinian population could be higher than that observed among patients who successfully reached our center. While some series report lower proportions, studies from resource-diverse networks, including the International Consortium on APL (IC-APL), have reported comparable or higher high-risk distributions, underscoring the impact of referral patterns and delays in presentation [9].

From a health-systems perspective, our findings reinforce that early death remains the main barrier to cure in APL and is concentrated in the first weeks after diagnosis. In Palestine, efforts should focus on rapid recognition of suspected APL, immediate initiation of ATRA at first suspicion, and strengthened transfusion and critical care support. Experience from IC-APL and the modified Mexican IC-APL report suggests that structured networking, standardized protocols, and targeted education can reduce early mortality and improve survival in developing settings [9, 23].

Differentiation syndrome was observed in 24.1% of patients and was observed in both ATO-based and chemotherapy-based induction. All documented cases responded to dexamethasone, and temporary interruption of ATRA was used when clinically indicated; however, grading of severity could not be performed due to incomplete documentation. In addition, cytoreductive strategies were used when leukocytosis was present or rising during induction. Hydroxyurea was preferentially given to patients with higher baseline leukocytosis and higher-risk disease, and its use was not associated with increased early death, although bleeding complications were numerically more frequent among patients receiving cytoreduction, likely reflecting confounding by baseline disease burden.

This study has several limitations. Its retrospective design and single-center setting limit generalizability and introduce a risk of selection and information bias. The sample size is small, with only four early-death events, which restricts statistical power and precludes robust multivariable analysis of factors associated with early death. Some clinical and laboratory data were missing because documentation was incomplete, and MRD monitoring by RT-PCR was not consistently available. Follow-up duration, although sufficient to capture early outcomes, may not fully reflect late relapses. In addition, our single-center retrospective design and referral constraints, including limited access for patients transferred from Gaza, may have introduced selection bias and led to underestimation of early mortality and the burden of high-risk disease.

Nonetheless, the study also has important strengths. To our knowledge, it represents the first systematic description of adult APL in Palestine and provides real-world data on presentation, treatment, supportive care, and outcomes from a key referral center. The cohort includes all consecutive adult cases treated over an 8-year period, and data on complications and supportive care were captured from routine clinical practice. Notably, the results suggest that, with timely diagnosis and implementation of ATRA-based protocols, outcomes in a resource-limited setting can approximate those reported in high-income countries.

### Strengths and limitations

APL is a hematologic emergency in which bleeding-related early death remains the main barrier to cure. In this single-center Palestinian cohort, ATRA-based treatment achieved high hematologic remission rates with no observed relapse during follow-up. As the first systematic report of adult Palestinian APL, this study adds real-world data from a resource-limited referral setting. Patients with early death had a higher leukocyte burden and longer aPTT at diagnosis. Strengthening early recognition, immediate ATRA initiation, and standardized supportive care may further reduce early mortality in Palestine.

Nonetheless, the study also has key strengths. It provides rare real-world data on adult APL presentation, treatment, supportive care, and outcomes from a major Palestinian referral center, a setting for which published evidence remains limited.

## Conclusions

APL is a hematologic emergency in which bleeding-related early death remains the main barrier to cure. In this single-center Palestinian cohort, ATRA-based treatment achieved high hematologic remission rates with no observed relapse during follow-up. This study adds real-world data from a resource-limited Palestinian referral setting where published adult APL data remain scarce. Patients with early death showed higher leukocyte burden and longer aPTT at diagnosis. Strengthening early recognition, immediate ATRA initiation, and standardized supportive care may further reduce early mortality in Palestine.

## Data Availability

The datasets used and/or analysed during the current study are available from the corresponding author on reasonable request.

## Abbreviations

APL: Acute promyelocytic leukemia
AML: Acute myeloid leukemia
ANC: Absolute neutrophil count
ATRA: All-trans retinoic acid
ATO: Arsenic trioxide
CR: Complete remission
DIC: Disseminated intravascular coagulation
DFS: Disease-free survival
ECOG: Eastern Cooperative Oncology Group
FISH: Fluorescence in situ hybridization
ISTH: International Society for Thrombosis and Haemostasis
IQR: Interquartile range
MRD: Minimal residual disease
MTX: Methotrexate
NNUH: An-Najah National University Hospital
PT: Prothrombin time
RT-PCR: Reverse transcriptase polymerase chain reaction
WBC: White blood cell count
6-MP: 6-mercaptopurine
